# Cyst nematode bio‐communication with plants: implications for novel management approaches

**DOI:** 10.1002/ps.6105

**Published:** 2020-10-13

**Authors:** Juliet Ochola, Danny Coyne, Laura Cortada, Solveig Haukeland, Margaret Ng'ang'a, Ahmed Hassanali, Charles Opperman, Baldwyn Torto

**Affiliations:** ^1^ International Centre of Insect Physiology and Ecology Nairobi Kenya; ^2^ Chemistry Department Kenyatta University Nairobi Kenya; ^3^ East Africa, International Institute of Tropical Agriculture Nairobi Kenya; ^4^ Department of Biology, Section Nematology Ghent University Ghent Belgium; ^5^ Norwegian Institute of Bioeconomy Research Ås Norway; ^6^ Department of Entomology and Plant Pathology North Carolina State University Raleigh NC USA

**Keywords:** behavior, cyst nematodes, pest management, physiology, RNAi, semiochemicals

## Abstract

Bio‐communication occurs when living organisms interact with each other, facilitated by the exchange of signals including visual, auditory, tactile and chemical. The most common form of bio‐communication between organisms is mediated by chemical signals, commonly referred to as ‘semiochemicals’, and it involves an emitter releasing the chemical signal that is detected by a receiver leading to a phenotypic response in the latter organism. The quality and quantity of the chemical signal released may be influenced by abiotic and biotic factors. Bio‐communication has been reported to occur in both above‐ and below‐ground interactions and it can be exploited for the management of pests, such as cyst nematodes, which are pervasive soil‐borne pests that cause significant crop production losses worldwide. Cyst nematode hatching and successful infection of hosts are biological processes that are largely influenced by semiochemicals including hatching stimulators, hatching inhibitors, attractants and repellents. These semiochemicals can be used to disrupt interactions between host plants and cyst nematodes. Advances in RNAi techniques such as host‐induced gene silencing to interfere with cyst nematode hatching and host location can also be exploited for development of synthetic resistant host cultivars. © 2020 The Authors. *Pest Management Science* published by John Wiley & Sons Ltd on behalf of Society of Chemical Industry.

## Graphical abstract

Graphical representation of abiotic and biotic factors influencing the hatching and host seeking stages of cyst nematodes and approaches for use of semiochemical‐ and RNAi‐based management tools.

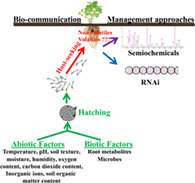

## 1 INTRODUCTION

Bio‐communication is a signal‐mediated interaction within and between living organisms. These signals include visual, auditory, tactile and chemical, and can function in single, binary and ternary interactions both above‐ and below‐ground in a wide range of organisms; animals, insects, plants, microorganisms among others.^1^, ^2^, ^3^ The most commonly studied form of bio‐communication is chemically‐mediated interactions between organisms; an emitter releases the chemical signal that is detected by a receiver leading to elicitation in a behavioral and/or physiological response in the latter organism. In cyst nematodes, which are soil‐borne pests, bio‐communication drives most of their life stages ranging from hatching to feeding site establishment, which if decoded can be exploited to manage these pests.

Cyst nematodes cause significant crop production losses worldwide. The most detrimental species of cyst nematodes belong to the genera *Globodera* and *Heterodera*, including potato cyst nematodes (*Globodera pallida* and *G. rostochiensis*), soybean cyst nematodes (*Heterodera glycines*), sugar beet cyst nematode (*H. schachtii*) and cereal cyst nematodes (*Heterodera avenae* and *H. filipjevi*) among others.^4^ Cyst nematode attack diverts host plant nutrients to the nematode and causes physical damage to the plant tissues as the nematodes migrate through the roots, leading to potential secondary infections.^4^ To induce feeding sites in roots, nematodes secrete effectors to alter the host cell structure. Additionally, they suppress host plant defences, which increases the susceptibility of the plant to attack by other pests and pathogens and reduce competition with weeds.^5^

Cyst nematodes attack essential crops, such as potatoes, soybeans, beans, rice, wheat, and barley, among others. Previously, it was thought that cyst nematodes existed only in temperate regions,^6^ but recent reports refer to indigenous populations in the tropics as well as alien, invasive species introduced from other parts of the world.^7^, ^8^ Increased human mobility and trade of agricultural produce appear to contribute to continued introduction of alien species in different parts of the world, including Africa where cyst nematodes are progressively becoming a problem.^2^ Losses due to cyst nematodes vary between regions while determining realistic estimations is challenging due to other factors that can additionally contribute to crop failure, including abiotic and biotic constraints. Indeed, much of the available data on the economic importance of cyst nematodes are over a decade old, as seen in a recent review.^6^ Details on the financial losses due to cyst nematodes in some regions is scarce, but it appears that their impact would be particularly heavy on smallholder farming systems, which dominate the African agricultural sector.

Cyst nematodes management methods include crop rotation, fallow, trap crops, organic soil amendments, solarization, intercropping, chemical nematicides, biological control agents and genetic resistance. These methods have been used with varying degrees of success, while each is subject to various limitations. For instance, some synthetic nematicides have high mammalian toxicity and pose an adverse environmental hazard, hence their withdrawal from markets.^9^ The effectiveness of resistant cultivars in a mixed population is limited by the species‐specific response of resistance genes to certain nematode species, permitting other parasitic nematode species/pathotypes to reproduce, when present. The repeated use of resistant cultivars can also lead to the rapid breakdown of resistance, and to the build‐up of resistance‐breaking nematode populations, as seen in soybean with *H. glycines*.^10^

**Figure 1:**
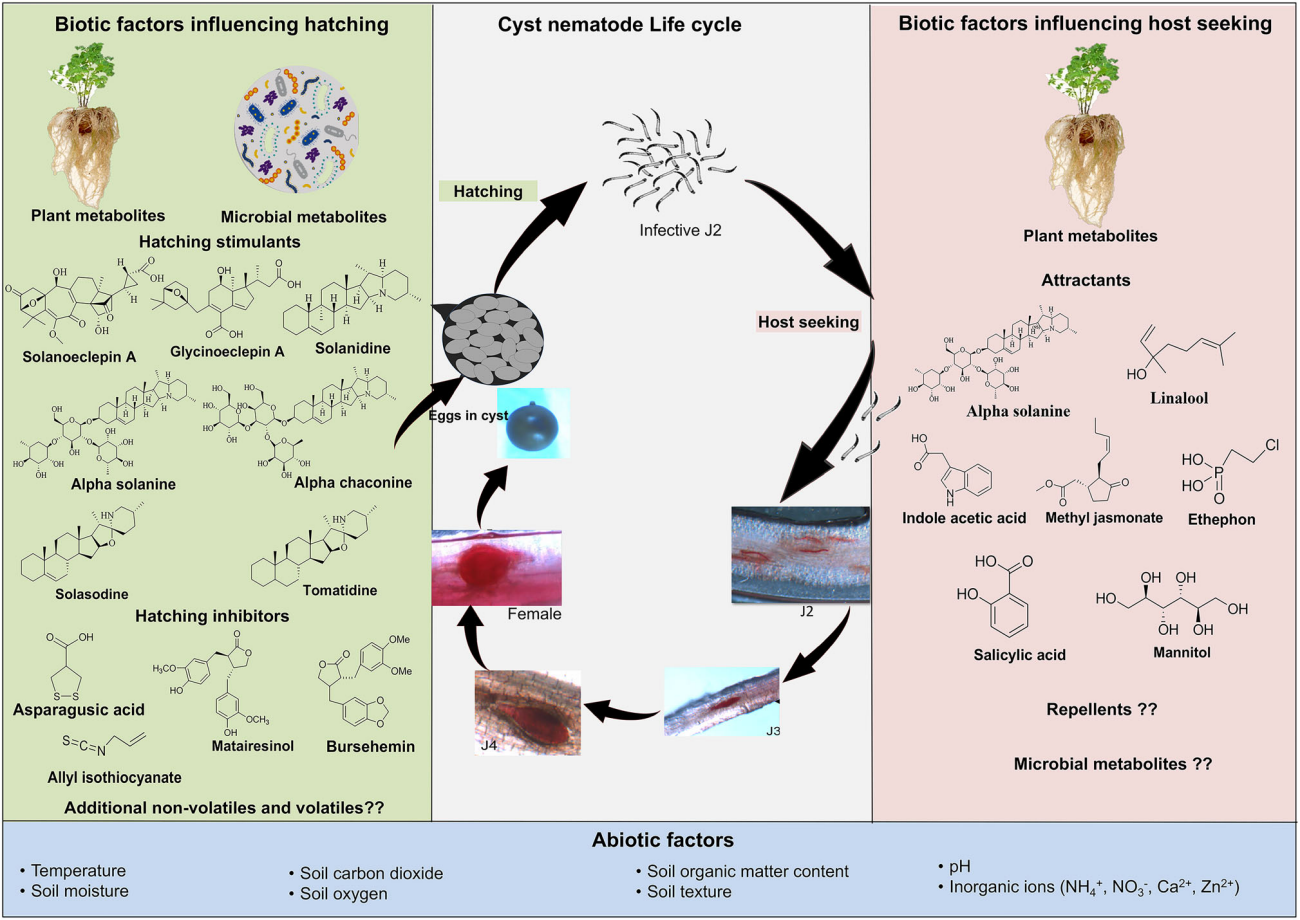
A schematic representation of cyst nematode life‐cycle including the abiotic and biotic factors influencing the hatching and host‐seeking phases.

To improve on the existing management strategies, it is critical to understand the abiotic and biotic factors, and the physiological mechanisms that influence bio‐communication in cyst nematodes. A recent review discussed the influence of root metabolites in the performance of soil nematodes.^11^ However, the review focused primarily on a polyphagous root knot nematode species without exploring how abiotic and biotic factors influence the bioactivity of these metabolites. Interestingly, cyst nematode hatching and the successful infection of hosts are biological processes that are largely influenced by semiochemicals (Fig. 1). These semiochemicals are produced either by the host plant or host‐associated microbes in the rhizosphere.^12^, ^13^ Antagonistic plants and microbes can also influence cyst nematode bio‐communication with host plants. Here, we review the literature on the abiotic and biotic factors influencing bio‐communication in cyst nematode hatching and host seeking stages and outline potential targets in their life cycles, for improved management in the field.

## 2 HATCHING AND HOST‐SEEKING PROCESS

Cyst nematode hatching may be simple or complex depending on the nematode species. A cyst can contain hundreds of eggs, which are surrounded by the cyst wall. The cyst wall is a robust but permeable structure that acts as an interface between the nematode and its external environment. The cyst wall of *G. rostochiensis* is composed of mainly proteins, lipids, carbohydrates, glucosamine, polyphenols and inorganic matter.^14^ The cyst wall also contains hatching inhibitors, such as protease and protease inhibitors, which are found in high concentrations during unfavorable conditions for cyst persistence in the soil.^2^, ^15^ Understanding the structure and composition of the cyst wall is therefore essential, since any management strategy intending to target the encapsulated juvenile stages must first interact with or penetrate the cyst wall, before reaching the eggshell and the juvenile.

The mechanism described in this section primarily uses *G. rostochiensis* as an example, a species that is almost entirely reliant on potato and tomato root diffusates to hatch. The first juvenile stage (J1) develops from an embryo, before molting into the J2 within the egg. Hatching of the J2 involves physico‐chemical alteration of the eggshell structure comprising three layers: outer lipoprotein layer, middle chitinous layer and an inner semi‐permeable lipid layer.^16^ Although permeable to respiratory gases, the lipid layer blocks the passing of water‐soluble molecules until its permeability is altered. Thus, unhatched eggs of *G. rostochiensis* usually occur in a state of partial hydration due to osmotic pressure generated by the disaccharide trehalose. Trehalose is found in the perivitelline fluid between the unhatched J2 and the eggshell and it protects the J2 against environmental shocks by inducing quiescence and inhibiting motility.^17^ High trehalose concentrations are found in the potato cyst nematode. This is to ensure that hatching occurs only in the vicinity of its host. Most cyst nematode species with a wider host range, like *H. schachtii*, have a lower osmotic pressure due to low trehalose concentration enabling eggs to readily hatch in water without the need for host cues.^16^, ^18^

In *G. rostochiensis*, a calcium‐mediated process alters the permeability of the eggshell, increasing signalling of hatching factors. This process terminates diapause in the eggs and allows for an influx of water into the egg to hydrate the J2. This initiates a series of metabolic changes within the egg.

Physiological studies have revealed that the hydrated J2s emerge from the egg through a slit in the eggshell, which they create by thrusts from their stylets, allowing them to exit the cyst through the vulval fenestra or anus.^16^, ^18^ However, in some instances hatched J2s may fail to emerge from the cyst. Although evidence indicates that this may depend upon the concentration of the root diffusate or any of the diverse classes of individual compounds that contribute to stimulating hatch and/or host location,^19^, ^20^ the mechanisms involved remain largely unknown. Recently, it has been proposed that additional chemical cues, including a feedback signal between the emerged J2s, combined with host root signals, may be required to stimulate optimal emergence and to complete the host location process of J2s.^19^ Alternatively, the sudden burst of emerged J2s in response to hatching stimuli may create overcrowding at the vulval fenestra, preventing J2 movement and exit, as well as increasing consumption of energy reserves, causing some J2s to remain encysted.^19^

After hatching, cyst nematode J2s rely on lipid reserves as their sole source of energy until they locate a host plant, penetrate its root, and establish the syncytium (feeding site) in the vascular cylinder. The J2s use chemical and tactile cues as primary signals for host plant detection, orientation, guidance and communication (*e.g*. sex pheromones) in the rhizosphere.^21^ Chemoreception in cyst nematodes is assumed to be perceived by external sensilla, comprising the anterior amphids which are the main olfacto‐sensory organ. The amphidal canal houses the neuronal dendrites that are bathed in secretions from the socket cells that may facilitate signal transduction. These secretions contain proteins that may play an important role in chemoreception.^18^, ^22^ Chemical stimuli come into contact with these secretions and reach the receptors through diffusion. The amphids contain multiple sensory neurons and variable secretory materials, making them selective and sensitive signal transducers.^18^, ^23^ For example, the sensory neurons of the nematode, *Caenorhabditis elegans*, have been shown to be multifunctional and each neuron bears more than one type of receptor.^24^ Chemoreception of the identified hatching factors is yet to be elucidated for cyst nematodes.

Hatched J2s are attracted to and locate host plants in response to root phytochemicals. Upon locating a host root, the J2 injects esophageal secretions containing polysaccharide degrading enzymes and cell wall modifying proteins including ß‐1,4‐endoglucanases, pectate lyases and expansins, which cause a series of metabolic transformations leading to the hypertrophy and partial degradation of the cell walls.^17^, ^25^ This also triggers several neighboring plant cells to undergo a similar process, which merge and form a multinucleate syncytium.^25^ J2s then molt into J3 and then J4 before developing into adults (Fig. 1). Whether they develop into either a male or female may depend upon a balance between the nutritional quality and interaction with plant defence compounds, and the nematode's ability to ingest nutrients, while suppressing defence compounds.^26^ The female nematode remains sedentary and feeds by extracting nutrients from the plant. This allows its body to swell, which increases its reproductive capacity, allowing it to break through the root surface while remaining attached to the roots. Males become vermiform (cylindrical and slender) and actively move out of the root to fertilize the sedentary females through sexual reproduction. Hundreds of eggs then begin to develop within the female, or in external egg sacs. Once the eggs mature, the female dies, and the outer cuticle hardens to protect the encysted eggs. The time taken to complete a life cycle varies among cyst nematode species, as well as depending on environmental conditions (moisture and temperature) and interaction between the nematodes and their hosts. Species such as *H. glycines* can complete several generations within a single soybean cropping season.^6^

## 3 ABIOTIC FACTORS INFLUENCING HATCHING AND HOST‐SEEKING

The parasitic behavior of cyst nematodes is a result of complex interactions among the host plants, nematodes, and environmental factors (Fig. 1). With increasing climate change, a clear understanding of how environmental factors influence cyst nematode‐host plant interactions can provide an insightful basis for the development of sustainable management strategies that can withstand rising temperatures and elevated CO_2_ levels.

Temperature has a profound effect on plant parasitic nematode development,^27^ and specific temperature ranges are required for various processes during the successive stages of the cyst nematodes life cycle. Depending on species, the effectiveness of the response to stimuli may be dependent on both the optimum temperature for hatch and geographic origin of the cyst nematode population. For instance, when given the same hatching stimulation, optimum hatch of *H. avenae*, originating from Egypt was, on average, 5 °C higher than a population originating from Germany, suggesting evolutionary adaptation to local conditions.^28^ Soil temperature studies have also shown that elevated temperatures deplete *G. rostochiensis* lipid reserves faster and likely result in increased rates and amounts of hatching for both potato cyst nematode species.^27^, ^29^

Soil texture, moisture, carbon dioxide and oxygen contents can also influence cyst hatching and J2 host‐seeking behavior. For example, maximum cyst nematodes hatching generally occurs when the soil moisture content is at field capacity.^18^ For *H. glycines*, optimal J2 hatching occurs at 25% soil moisture content but this declines by about 75% following a 10% decrease in moisture content.^30^ Drought and waterlogging tend to hinder hatching.^18^ Likewise, high soil humidity beyond field capacity also reduces the rate of *H. glycines* and potato cyst nematode hatching, thus increasing nematode mortality due to a reduction in the oxygen concentration in the soil.^31^ These findings suggest that depending on the moisture conditions, cyst nematodes may be exposed to higher or lower concentrations of water‐soluble hatching stimulants and phyto‐attractants. Increased carbon dioxide levels and short‐chain fatty acid content in the soil, derived from available organic matter, inhibit potato cyst nematode hatching.^32^ Fluctuating oxygen concentrations can reduce hatching rates of *H. schachtii*.^33^ On the other hand, aeration results in increased hatching of *H. avenae* and *G. rostochiensis*.^33^ These findings indicate that soil gas exchange and organic matter quality and quantity are important in modulating cyst nematode bio‐communication, and potentially their physiology. Additionally, hatching factors and phyto‐attractants may interact with soil texture. Higher natural decline for potato cyst nematode occurs in sandy soil than in peaty and clay soils. *G. pallida* specific hatching factors have a lower affinity to soil matrices, than those of *G. rostochiensis*, suggesting a greater potential to control *G. pallida* through suicidal hatch than *G. rostochiensis*.^34^ Coarse‐textured sandy soil and associated pore space are most suitable for nematode migration. For example, soil particle sizes of between 150–250 μm is most suitable for movement of *H. schachtii*,^35^ which can influence the nematode hatching process, migration to and invasion of roots, and diffusion of hatching factor and volatile phyto‐attractants. Additionally, pH levels greater than 8.0 can rapidly inactivate hatching factors.^18^ However, one study has shown that potato root diffusate pH has no detrimental effect on the chemoattraction of *G. pallida* although this study did not test pH above 7.5.^36^

Inorganic ions and salts present in the soil can also stimulate or inhibit hatch of cyst nematodes. The inorganic ions ammonium or nitrate, commonly found in fertilizers, inhibit *H. glycines* hatch,^37^ while calcium sulphate and zinc ions at concentrations simulating natural soil levels stimulate hatching.^38^ High organic matter content in the rhizosphere tends to decrease the mobility of hatching factors and interference with J2 mobility.^34^, ^39^ These observations suggest that plant roots growing in soils with low organic matter become more easily detected and located by J2. For example, field studies revealed that *G. rostochiensis* hatching activity occurred 4 weeks after potato root diffusate application in fields with 2% soil organic content, while in fields with 62% organic content no hatching was observed.^34^ A better understanding of the abiotic factors that influence J2 hatching, host seeking and migration will help in developing more effective nematode management approaches.

## 4 BIOTIC FACTORS INFLUENCING HATCHING AND HOST‐SEEKING

### 4.1 Hatching stimulants

Plants secrete root diffusates into the rhizosphere, creating varying interactions with the root microbiome. Root diffusates contain a range of organic compounds that may function as semiochemicals (attractants, repellents, hatching stimulants or hatching inhibitors) among other functions within the rhizosphere (Fig. 1). Cyst nematodes utilize these compounds as cues for parasitic behavior and synchronize their life cycle with that of the plant. The composition of root diffusates varies both qualitatively and quantitatively depending upon the plant species, cultivar, plant developmental stage, and microbial presence. Even though most cyst nematodes are strongly reliant on hatching factors in host root diffusates to hatch, a few species readily hatch in water.^18^ Nonetheless, the presence of host root diffusates can enhance the hatching rate of most cyst nematodes species.^18^ This response points to a plant‐pest communication strategy to ensure or optimize hatching in the presence of a suitable host, thus maximizing its survival.

Root diffusates from young plants tend to have a higher stimulatory effect on hatching than those from more aged/mature plants, attributed to either variations in the composition or concentration of the root diffusates. This demonstrates an adaptation of cyst nematodes to perceive the most suitable vegetative phase of host plants for optimum infection and survival.^18^ The decline in potato cyst nematode hatching in the presence of mature plants could be due to change in the photosynthate partitioning (*e.g*. flowering, tuber formation) leading to a quantitative or compositional change in root diffusates. The stimulatory effect of root diffusate on hatching is dose‐dependent, often with optimal stimulation achieved at relatively low doses. At high doses, root diffusates can cause supra‐optimal inhibition, which is an intrinsic characteristic of hatching factor‐receptor interaction at high ligand concentration. Low doses of root diffusates resulting in high levels of hatch stimulation make a promising and practical target for use in exogenous application stimulation of ‘suicidal hatching’. ‘Suicidal hatching’ is the stimulation of cyst nematode hatching that result in eventual death of the nematode due to the absence of host plants. This can be achieved in various ways such as use of trap crops that can stimulate hatching but which do not allow nematode development and reproduction; exogenous application of host root diffusates; and use of synthetic hatching factors that do not allow hatched J2s to migrate from the cyst.

The changing vegetative state of a plant during growth can also influence cyst nematode dependency on host root diffusates for hatching. For example, hatching of pigeon‐pea cyst nematode, *H. cajani*, encysted eggs during a growing period was similar over the first four generations, but in the fifth and sixth generations, hatching was highly dependent on host root diffusates.^18^ This demonstrates yet another cyst nematode adaptation to ensure survival in the absence of the host.

Some of the host derived hatching factors include polar compounds: the pentanortriterpene glycinoeclepin A, isolated from the root extract of the kidney bean (*Phaseolus vulgaris*) stimulates hatching of *H. glycines*,^11^, ^16^, ^18^ the tetranortriterpene solanoeclepin A, isolated from tomato (*Solanum lycopersicum*) and potato (*Solanum tuberosum*) root diffusates stimulates hatching of potato cyst nematodes (Table S1).^11^, ^16^, ^18^, ^40^ Other host specific hatching factors include glycoalkaloids α‐solanine and α‐chaconine, steroidal alkaloids solanidine, solasodine and tomatidine for *G. rostochiensis*, and monosaccharide glucose and fructose for *G. pallida* isolated from potato (Table S1).^19^, ^41^ Root diffusates contain multiple hatching factors that exhibit a synergistic effect with one another, as seen in purified isolates stimulating lower hatch compared to the crude.^42^ The presence of about nine *G. rostochiensis* hatching factors, all closely related structurally (same molecular mass and similar mass fragmentation patterns), in potato root diffusates was detected even though the structures of these hatching factors were not identified.^42^ This differential hatching response of *G. rostochiensis* to these hatching factors^42^ demonstrates the specificity of the nematode to hatching factors and thus the need to better understand the nature of the hatching receptors.

Interestingly, root diffusates of some non‐host plant species also stimulate cyst nematode hatch, which suggests that such plants can be exploited as either candidates for ‘suicidal hatching’ using synthetic compounds or act as ‘dead‐end’ trap crops to reduce cyst nematode densities (Table S1). Resistant and susceptible cultivars of the same host plant species appear to show no correlation with hatch induction,^43^ which indicates that resistance‐conferring genes that affect root diffusate activity have yet to be found. Compounds stimulating hatching have also been detected in *G. rostochiensis* and *H. glycines* cyst wall and egg homogenates and rinsates,^44^, ^45^ however, their identities are yet to be established. Other synthetic compounds (Table S1) and inorganic ions have also been found to stimulate hatch of cyst nematodes.

Although several naturally occurring hatching factors are known, most are yet to be tested for cyst nematode management. The complexity of these molecules makes their synthesis expensive and precludes large‐scale production. However, there is evidence that there are yet more potent potato cyst nematode hatching factors, such as those detected in the root diffusates of potato and *Solanum sisymbriifolium* stimulating hatching of *G. pallida*, although the main PCN hatching factor solanoeclepin A was not detected in the root exudate chemical profiles.^42^, ^43^ Moreover, previous studies on cyst nematodes hatch stimulation have concentrated on water‐soluble non‐volatile compounds. Host plant roots also release volatile organic compounds that can diffuse in the soil and influence nematode behavior.^46^ It would be interesting to investigate the role of these constituents on cyst nematode hatching.

### 4.2 Hatching inhibitors

The modes of action of the hatching inhibitors are not well understood, even though they are thought to either inactivate the hatching factor or competitively bind to the hatching factor‐receptors in the eggshell. High production of hatching inhibitors in host root diffusates like potato occurs in the initial weeks of growth. This could be to prevent young, immature plants from being over‐challenged with potato cyst nematode infection at an early stage.^34^ The identities of these hatching inhibitors are yet to be elucidated. Root diffusates and extracts from non‐host plant species from the Apiaceae and Brassicaceae families have also been implicated in inhibiting hatching of cyst nematodes (Table S1). Several analogues of glycinoeclepin A, inhibited hatch of *H. glycines* and the active component for the inhibition was identified as a keto diacid derivative.^47^ Although much progress has been made towards elucidating natural hatching factors/inhibitors from plant root diffusates, further research efforts in this area are warranted given their potential for downstream development.

### 4.3 Attractants and repellents

The specific signals responsible for cyst nematode attraction to host plants remain largely unknown. Chemical signalling involved in cyst nematodes host‐seeking is a complex of interactions that include both general and host‐specific cues. Since cyst nematode hatching occurs in the vicinity of the host plant, long‐distance attractants more likely act as general cues that help orient the nematode to the rhizosphere.^18^ Once J2 approach the roots, they use concentration gradients of short‐distance attractants, which are mainly non‐volatile water‐soluble compounds present in the host plant root, to enable them to locate the root.^21^ For instance, as reported in a recent review *G. pallida* is attracted to root phytochemicals including α‐solanine, linalool, indole acetic acid, ethephon, salicylic acid, methyl jasmonate and mannitol (Table S1).^11^, ^48^, ^49^ Bulb extracts of *Narcissus tazetta* significantly reduced *H. glycines* J2 motility and attraction to soybean root tip,^50^ while root diffusates and extracts from marigold, soybean, and pepper were attractive.^48^, ^51^ However, the identities of the mediating compounds remain unknown. Identification of these compounds can provide opportunities for genetic manipulation to reduce J2 attraction and infection. Non‐host plants can be used as intercrops or their plant parts with bioactive properties used as extracts in powder or cake form as soil amendments as reported for *N. tazetta* in the control of *H. glycines*. Establishing the performance of these crops/amendments under field conditions is also warranted.

The ethylene signaling pathway is also involved in influencing and modulating the attraction of parasitic nematodes to host plant roots,^52^, ^53^, ^54^ although whether this signaling regulates the production of the attractants or is directly involved remains to be fully established. Ethylene signaling reduces *H. glycines* attraction to host roots,^52^, ^55^ unlike with *H. schachtii* for which ethylene signal transduction increases plant susceptibility.^53^ Disruption of strigolactone signaling also compromises *H. schachtii* attraction to *Arabidopsis* roots.^56^ ABC transporter genes modulate composition of root diffusates with knockdown of ABC‐G33 and ABC‐C6 in tomato, resulting in hatch inhibition and repulsion of both *G. rostochiensis* and *G. pallida*.^57^ It is clear, therefore, that there are numerous chemical cues that are yet to be discovered in the cyst nematode‐host relationship, and that we have yet to fully understand the role of those already identified.

Most studies on chemoattraction of cyst nematodes, have focused on water‐soluble non‐volatile compounds.^58^ Volatile compounds can diffuse over long distances in the soil, making them excellent candidates as long‐distance attractants and for potential exploitation in management. This has been demonstrated with root‐produced volatile compounds associated with *Meloidogyne incognita* attraction to host plant roots.^46^ The role of volatile compounds in chemoattraction of the *G. pallida*,^36^ has already been highlighted and as such, further investigations are warranted.

Plants have also developed defensive mechanisms to counter cyst nematode attacks using chemical repellents.^27^ Production of repellent compounds in root diffusates has been demonstrated in fractions of potato root diffusates that elicited deterrence to *G. rostochiensis* and *G. pallida*.^58^ *H. schachtii* J2s were also repelled by roots of oat. Focus on repellent compounds may, therefore, prove as useful as attractants in the quest for developing cyst nematode management options. Thus, further research covering a wider range of cyst nematode species, identities of the repellent compounds and verification of efficacy under field conditions is required.

### 4.4 Role of microorganisms

There is increasing evidence revealing the role of microbes in cyst nematode hatching and host‐seeking.^18^, ^22^, ^59^ Several studies have demonstrated microorganism positive involvement in the spontaneous hatching of potato cyst nematode in the field in the absence of host plants.^60^ Given the complex nature of smallholder farming systems in Africa, (mixed cultivation of a range of plants in a multilayer tapestry of unsystematic randomized mixed cropping),^10^ an exploration of the microbiome of plants and how they affect cyst nematodes would be a start for developing efficient biological control. Bioactive microorganism can either be directly used to treat fields through microbiome transfer from nematode suppressive soils or isolation of their bioactive compounds to treat fields where the microorganisms are not able to survive.^13^, ^22^ For example, use of the bio‐nematicide from the fermentation of the soil hyphomycete fungus, *Myrothecium verrucaria*, which irreversibly prevents hatching of potato cyst nematode.^61^ Other microbial isolates and culture filtrates directly affecting cyst nematode hatching are provided in Table S1.

Plant root exudates can be transformed by microorganisms colonizing the rhizosphere, resulting in a qualitative and quantitative alteration of root diffusates. Potato plants inoculated with arbuscular mycorrhizal fungi stimulated the production of *G. pallida*‐selective hatching factors or hatching factor stimulants, increasing hatching compared to uninoculated plants.^59^ Root diffusates from plants cultivated under non‐sterile conditions contained hatching factors of potato cyst nematodes that facilitated 57% more hatching activity compared to root diffusates of aseptically cultivated plants.^62^ These findings confirm the involvement of host plant rhizosphere microorganisms in cyst nematode hatching to increase the number of hatching J2s and to maximize infections. This hatching response can also be an indication of survival of other microbes in the rhizosphere, which in turn can influence the nematode's own survival.

Microbe produced compounds can also influence cyst nematodes chemotaxis response, as found with *H. schachtii* J2 attraction to bacteria isolated from sugar beet rhizosphere and *H. glycines* repulsion from soybean plant in the presence of cultural supernatant from *Sinorhizobium fredii*.^13^ Microbial volatile organic compounds have also been shown to influence interactions within the rhizosphere^63^ with some possessing nematicidal, fungicidal, plant growth‐promoting/‐inhibiting activities. Studies on the effect of microbial volatile organic compounds on plant parasitic nematodes have, to date, focused mainly on their effect on root knot nematodes, *Meloidogyne* spp., which demonstrates both our limited understanding of these compounds and an indication of their potential as a management tool for cyst nematodes.^64^

## 5 BIO‐COMMUNICATION FACTORS FOR THE NEMATODES MANAGEMENT

### 5.1 Semiochemicals

**Figure 2:**
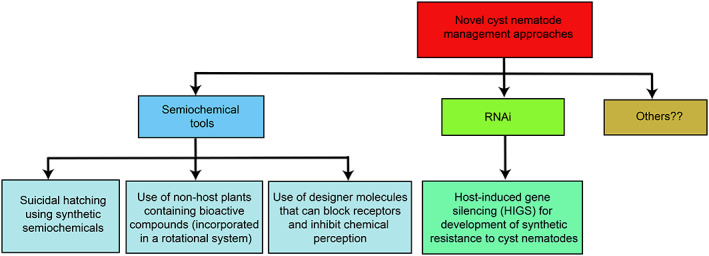
Novel approaches in the management of cyst nematodes.

Suicidal hatching can be triggered in cyst nematodes with root diffusate phytochemicals. This is demonstrated by the application of tomato root diffusates in *G. pallida* infested fields to achieve 69–79% reduction in viable eggs in a cyst after 12 weeks.^34^ Although effective, the availability of root diffusates to achieve this effect could be a challenge. Isolation of specific hatching factors from the host root diffusates has also proven to be very challenging, especially due to the minimal amounts of hatching factors produced by plants. Furthermore, natural hatching factors, such as glycinoeclepin A and solanoeclepin A are very expensive to synthesize making them impractical for field use. A recent study has shown that of the several classes of compounds identified in potato root diffusates, only glycoalkaloids, α‐chaconine and α‐solanine and steroidal alkaloids solanidine, solasodine and tomatidine stimulated *G. rostochiensis* hatching.^19^ Because similar compounds are present in certain potato cyst nematode non‐host plants, such as the edible African indigenous nightshade species *Solanum villosum*, *S. nigrum*, *S. scabrum*, *S. sarrachoides*,^65^, ^66^ this opens opportunities for use of such plants either in a rotation system, as organic amendments, trap crops, cover crops or as a processed‐plant‐product for management of potato cyst nematodes (Fig. 2). Non‐host plants producing repellent compounds can also be used as intercrops to mask the chemical communication between cyst nematodes and their hosts to cause a reduction in nematode infection and reproduction.

Blocking of the sensory perception of sedentary endoparasitic nematodes can been used to interfere with their host‐seeking ability, leading to depletion in energy reserves before the nematode can invade the host plant.^18^ Chemical disruption of cyst nematodes can be achieved by blocking the amphids, which are the main chemoreception organs.^18^, ^67^ Electrophysiological studies have demonstrated that exposure of *G. rostochiensis* to a commercial nematicide produced from fermentation of the fungus *M. verrucaria* prevented chemoreception of potato root diffusates, indicating that some of the microbial‐derived compounds can block the amphidal pores, or bind to amphidal secretions to interfere with nematode sensory perception.^68^ Zinc sulphate, which is a common constituent in most fertilizers also blocks chemoreception in *G. rostochiensis*.^69^ Further studies to determine optimal concentrations to achieve chemoreception blockage under field conditions is warranted. This could lead to the development of fertilizers with both plant growth promoting and chemoreception disruption effects. Identification of nematode chemosensory/hatching receptors and designing molecules that target these receptors can offer ways of inhibiting hatching and J2 host‐seeking processes (Fig. 2). Transgenic plants expressing chemo‐disruptive peptides can minimize invasion and enhance host plant resistance as recorded in the disorientation of invading *G. pallida* by transgenic potato plants expressing a secreted peptide that inhibit nematode acetylcholinesterase and nicotinic acetylcholine receptors.^70^ Similar to cyst nematodes, several studies have proved that semiochemicals can also be applied in the management of other plant parasitic nematodes including the polyphagous *Meloidogyne* species.^1^, ^2^, ^52^ For *Meloidogyne* species, targeting the host seeking stage with application of attractants, repellents and receptor blocking molecules to inhibit signal transcription or transduction offer much promise.

### 5.2 RNA interference (RNAi)

RNA interference (RNAi) was first reported to occur in the free‐living nematode *C. elegans* ^71^ and since the initial report of RNAi of plant‐parasitic nematode genes in the cyst nematodes *H. glycines* and *G.pallida*,^72^ it has been extended to numerous other genera.^73^, ^74^, ^75^ Remarkably, plant parasitic nematode genes appear to be uniquely susceptible to RNAi, with a very high degree of successful gene knockdown reported. The relatively few plant parasitic nematodes genes that are refractory to RNAi include some housekeeping genes, certain effectors, and several genes involved in the RNAi pathway itself.^74^, ^76^, ^77^, ^78^, ^79^ The reasons for this phenomenon remain elusive but functional redundancy of essential processes may have a role. It is therefore imperative to experimentally validate each candidate gene. The development of RNAi for plant parasitic nematodes has revolutionized approaches to determining gene function and has enabled reverse genetics to be applied. Given that genetic transformation has not yet been achieved for plant parasitic nematodes, RNAi reverse genetics represents a powerful tool to study gene function.

Because cyst nematodes rely heavily on semiochemicals from host plants for cues influencing hatching and host‐seeking behavior, RNAi approaches to suppressing gene transcription in signal reception and transduction present powerful discovery tools in defining these interactions. RNAi has recently been utilized to define potential functions of novel cyst nematodes effectors,^80^, ^81^, ^82^ supporting the idea that genes identified as important by their RNAi‐phenotypes may represent candidates for development of host‐induced gene silencing.^83^, ^84^ Host‐induced gene silencing has become the most commonly applied method to develop synthetic resistance to plant parasitic nematodes. A rapid screening approach was developed exploiting *Agrobacterium rhizogenes* hairy root transformation and the gateway cloning system for rapid screening of candidate genes,^85^, ^86^, ^87^ and various plant parasitic nematode genes involved in essential cellular functions have been used to achieve varying degrees of transient resistance.^85^, ^88^, ^89^, ^90^, ^91^ Genes providing plant parasitic nematode resistance in these screens are then amenable to generation of stable transformed plants through conventional means. The advent of technologies like gene stacking makes it possible to enhance host‐induced gene silencing approaches to plant‐parasitic nematodes. Reliance upon single‐gene technologies in the past has resulted in statistically significant plant nematode suppression, but at levels not acceptable economically to producers. As more genes are identified as crucial in the nematode parasitic cycle, more targets for host‐induced gene silencing stacking will be available.

Up‐regulated genes during hatching represent potential targets for RNAi to determine their functional roles in recognition of semiochemical signals and cellular processes involved in hatching. Candidates for RNAi screening for a reduced or suppressed hatching phenotype include the enzymes neprilysin (a zinc metalloprotease), and chitinase, identified as cellular process components in previous studies. In *C. elegans* and potato cyst nematodes, neprilysins are strongly expressed prior to hatching. There are over 20 neprilysin genes in *C. elegans*, while 11 different transcripts were up‐regulated in potato cyst nematodes in response to hatching factor. Homologs of both lap (*lap‐1*) and chitinase (*cht‐2*) have been identified in the potato cyst nematodes genome using *C. elegans* queries. Among the genes reported to be up‐regulated by hatching factor‐stimulus, many are components of calcium signaling and transport pathways, including *gcy‐9* (guanylate cyclase/peptide receptor), *mua‐3* (calcium ion‐binding activity), a gene encoding a DEL protein (four domain‐type voltage‐gated ion channel alpha‐1), and numerous transmembrane transporter genes^17^ Given that calcium plays a critical role in potato cyst nematodes hatching, these genes represent attractive candidates for functional characterisation by RNAi, as well as being potential host‐induced gene silencing constructs.

Based on extensive studies of the chemosensory and mechanosensory mechanisms of *C. elegans*, much is known about the neurobiology of these systems in free‐living nematodes. As there is limited knowledge on which neurosensory systems in plant parasitic nematodes play a role in host recognition, identification of homologous systems using *C. elegans* as a guide is a suitable starting point. The chemosensory system in *C. elegans* is catalogued by function (water‐soluble chemicals, volatile chemotaxis, *etc*.), with genes grouped as receptors, signal transduction, and regulators.^92^ A limited survey of available plant parasitic nematodes genomes using *C. elegans* protein sequences as queries reveals that the cyst nematodes possess homologs to many chemosensory genes. These include *gpa‐3* and *odr‐3* (receptors for water‐soluble and volatile chemotaxis, respectively), *tax‐2* and *tax‐4* (signal transduction), and *osm‐9* and *egl‐4* (regulators). Indeed, RNAi knockdown of *odr‐1* and *odr‐3* receptors and *tax‐2* and *tax‐4* signal transducers resulted in defective chemotaxis to volatiles, non‐volatiles, and host root exudates in *Meloidogyne incognita*, supporting a role in host recognition for these chemosensory genes.^93^ In addition to chemosensory gene homologs, cyst nematodes possess mechanosensory genes related to *mec‐10*, *mec‐4*, *trp‐1*, and *trp‐2* from *C. elegans*. Taken together, these genes represent excellent targets in cyst nematodes for RNAi to begin the unravelling of sensory mechanisms involved in host location/recognition by knockdown phenotype. Unlike *C. elegans*, where neuronal genes are mostly refractory^94^ RNAi of plant parasitic nematode neuronal genes is highly successful and repeatable, making this approach ideal for identifying key components of plant parasitic nematode sensory systems in response to host semiochemicals.^95^ The abundance of plant parasitic nematode RNAi tools have enabled high‐throughput screening of candidate genes listed above. Selection of suitable phenotypes for further development for host‐induced gene silencing approaches could result in synthetic resistant host cultivars *via* interference with cyst nematodes hatching or host location.

## 6 FUTURE PERSPECTIVES

Having a more comprehensive knowledge of the chemical constituents of root diffusates, especially the volatiles, will undoubtedly lead to our better understanding of the chemo‐ecological interactions between nematodes, their microbiome, and host plants. Clearly, there remains much to be established, in terms of the chemical signaling between cyst nematodes and their hosts. Chemical cues that play a role within rhizosphere also appear far more numerous and complex than we have so far anticipated and so, by fully characterizing the molecules involved in this chemical dialogue we will be better able to exploit this communication to disrupt specific signal pathways, especially when combined with host‐induced gene silencing approaches. This could be designed to either minimize attraction of host roots to nematodes or repel them. Alternatively, this approach can provide a mechanism to stimulate hatching in the absence of the host, or even to attract nematodes away from host roots, to non‐hosts or trap crops. Such environmentally sustainable approaches to the management of cyst nematodes, as well as other plant parasitic nematodes, offer enormous potential towards improving sustainable crop production. However, it is important that future efforts examine and exploit the chemical communication between soil organisms and plants and should take a multi‐disciplinary approach, including, amongst others breeding, biotechnology, plant pathology, as well as chemical ecology.

## CONFLICT OF INTEREST

The authors declare that the research was conducted in the absence of any commercial or financial relationships that could be construed as a potential conflict of interest.

## Supporting information


**Table S1.** Biotic factors influencing cyst nematode hatch
