# Supplementary material for: Cyst nematode bio‐communication with plants: implications for novel management approaches
Source: Pest Manag Sci. 2020 Oct 13;77(3):1150–9. doi: 10.1002/ps.6105 (PMC7894489; doi:10.1002/ps.6105)
Supplement: Supplementary file 1 — Table S1. Biotic factors influencing cyst nematode hatch [file PS-77-1150-s001.docx]

**Cyst Nematode Bio-communication with Plant: Implications for Novel Management Approaches**

**Running title: Bio-communication in Cyst Nematodes**

Juliet Ochola^1,2^, Danny Coyne^3,4^_,_ Laura Cortada^3,4^, Solveig Haukeland^1,5^, Margaret Ng’ang’a^2^, Ahmed Hassanali^2^, Charles Opperman^6^ and Baldwyn Torto^1^*****

**Table S-1: Biotic factors influencing hatching and host seeking of cyst nematodes**

| **Hatching stimulants** | | | | | |
| --- | --- | --- | --- | --- | --- |
| **Nematode species affected** | **Compound** | | **Class of compound** | **Source** | **Reference** |
| *Heterodera. glycines* | Glycinoeclepin A | | Pentanortriterpenoid | Kidney bean (*Phaseolus vulgaris*) root extracts | ^1-3^ |
|  | - | | - | Red clover (*Triforium platense*) | ^4^ |
|  | 1,10-phenanthroline | | Ligand | Synthetic chemical | ^5^ |
|  | Tannic acid | | Tannin | Synthetic chemical | ^6^ |
|  | - | | - | Root exudates of ryegrass (*Lolium multiflorum*) | ^7^ |
|  | - | | - | *H. glycines* cyst wall and egg homogenates and rinsates | ^8^ |
| *Globodera. rostochiensis* | Solanoeclepin A | | Tetranortriterpenoid | Potato (*Solanum* *tuberosum*) and tomato (*Solanum lycopersicum*) root diffusates | ^9^ |
|  | α-Solanine and α-Chaconine | | Steroidal glycoalkaloids | Potato (*Solanum* *tuberosum*) root diffusates | ^10, 11^ |
|  | Solanidine, Solasodine and Tomatidine | | Steroidal alkaloids | Potato (*Solanum* *tuberosum*) root diffusates | ^11^ |
|  | **-** | | **-** | *G. rostochiensis* cyst or egg homogenates | ^12^ |
|  | **-** | | **-** | *Oxalis tuberosa* and *Lupinus mutabilis* | ^13, 14^ |
|  | **-** | | **-** | *Solanum sisymbriifolium* | ^15-17^ |
| *G. pallida* | Solanoeclepin A | | Tetranortriterpenoid | Potato (*Solanum* *tuberosum*) and tomato (*Solanum lycopersicum*) root diffusates | ^9^ |
|  | Glucose and Fructose | | Carbohydrate | Potato (*Solanum* *tuberosum*) root diffusates | ^18^ |
|  | - | | - | *Oxalis tuberosa* and *Lupinus mutabilis* | ^13, 14^ |
|  | **-** | | **-** | *Solanum sisymbriifolium* | ^15-17^ |
| **Hatching inhibitors** | | | | | |
| *H. glycines* | | Asparagusic acid | Dithiolane | *Asparagus officinalis* root extract | ^19^ |
|  |  | - | - | *Sinorhizobium fredii* cultural filtrates | ^20^ |
|  |  | Allyl isothiocyanate | Isothiocyanate | Synthetic chemicals / degradation of allyl glucosinolates from *Brassica* plants | ^21^ |
| *H. schachtii* | | Allyl isothiocyanate | Isothiocyanate | Synthetic chemicals / degradation of allyl glucosinolates from *Brassica* plants | ^21^ |
|  |  | **-** | **-** | *Agrobacterium radiobactor* | ^13^ |
| *G. rostochiensis* | | **-** | **-** | Root diffusates from *Anthriseus silvestris, Arehangeliea litoralis, Pimpinella. major, Pimpinella saxifrage, Heracleum sibirieum* | ^22^ |
|  |  | Asparagusic acid | Dithiolane | *Asparagus officinalis* root extract | ^19^ |
|  |  | Tannin | Tannin | Chestnut tannin solution | ^6^ |
|  |  | (*E*)-Chalcone | Flavonoid | Synthetic chemical | ^23, 24^ |
|  |  | - | - | *Stenotrophomonas maltophilia* and *Chromobacterium* sp. | ^25^ |
|  |  | - | - | Fermentation of *Myrothecium verrucaria* | ^26^ |
|  |  | - | - | Root diffusates from tomato plants with the ABC-G33 and ABC-C6 transporter gene knocked down | ^27^ |
| *G. pallida* | | Bursehemin and Matairesinol | Lignans | Leaf extracts of *Bupleurum salicifolium* | ^19^ |
|  |  | (*E*)-Chalcone | Flavonoid | Synthetic chemical | ^23, 24^ |
|  |  | Allyl isothiocyanate | Isothiocyanate | Oil of black mustard | ^28^ |
|  |  | - | - | *Agrobacterium radiobactor* | ^13, 29^ |
|  |  | - | - | Fermentation of *Myrothecium verrucaria* | ^26^ |
|  |  | - | - | Root diffusates from tomato plants with the ABC-G33 and ABC-C6 transporter gene knocked down | ^27^ |
| *H. filipjevi* | | - | - | *Bacillus cereus* and *Achromobacter xylosoxidans* | ^30^ |
| **Attractants** | | | | | |
| *H. glycines* | | **-** | **-** | Root diffusates and extracts from marigold, pepper, or soybean seedlings | ^31^ |
| *H. schachtii* | | **-** | **-** | Metabolites of ethylene pathway in Arabidopsis thaliana | ^32^ |
| *G. rostochiensis* | | α-Solanine, | Glycoalkaloids | Synthetic compounds | ^33^ |
|  |  | - | - | Volatile compounds from potato root exudates | ^34^ |
|  |  | Linalool | Monoterpenoid | Synthetic compounds | ^35^ |
| *G. pallida* | | Indole acetic acid | Auxin/ Phytohormone | Synthetic compounds | ^36^ |
|  |  | Ethephon | Organic phosphorus compound/plant growth regulator | Synthetic compounds | ^36^ |
|  |  | Salicylic acid | Organic acid/ Phytohormone | Synthetic compounds | ^36^ |
|  |  | Mannitol | Carbohydrate | Synthetic compounds | ^36^ |
|  |  | Methyl jasmonate | Oxylipins | Synthetic compounds | ^36^ |
|  |  | α-Solanine | Glycoalkaloids | Synthetic compounds | ^33^ |
|  |  | Linalool | Monoterpenoid | Synthetic compounds | ^35^ |
|  |  | - | **-** | Volatile compounds from potato root exudates | ^37^ |
| **Repellents** | | | | | |
| *H. glycines* | | **-** | **-** | Bulb extracts of *Narcissus tazetta* | ^38^ |
|  |  | **-** | **-** | Metabolites of ethylene pathway in Soybean and Arabidopsis thaliana | ^39^ |
| *H. schachtii* | | **-** | **-** | Strigolactone signaling in Arabidopsis thaliana | ^40^ |
| *G. rostochiensis* | | **-** | **-** | Root diffusates from tomato plants with the ABC-G33 and ABC-C6 transporter gene knocked down | ^27^ |
| *G. pallida* | | **-** | **-** | Root diffusates from tomato plants with the ABC-G33 and ABC-C6 transporter gene knocked down | ^27^ |

**References**

1. Sikder MM and Vestergård M, Impacts of root metabolites on soil nematodes. *Front Plant Sci.* **10**: 1792-1792 (2020).

2. Masler E and Perry R, Hatch, survival and sensory perception Cyst Nematodes, ed. by Perry RN, Moens M, Jones JT, C.A.B.I, Wallingford, UK, pp. 44-73 (2018).

3. Bohlmann H, Introductory chapter on the basic biology of cyst nematodes, in Advances in Botanical Research, ed. by Escobar C, and Fenoll C, Elsevier 73: 33-59 (2015).

4. Atsuhiko Kushidal, Taketo Ueharal and Momotal Y, Effect of red clover on hatching and population density of *Heterodera glycines* (Tylenchida: Heteroderidae). *JPN J Nematol*  **32**: 69-76 (2002).

5. Nonaka S, Katsuyama T, Kondo T, Sasaki Y, Asami T, Yajima S, et al., 1, 10-Phenanthroline and its derivatives are novel hatching stimulants for soybean cyst nematodes. *Bioorg Medicinal Chem Lett* **26**: 5240-5243 (2016).

6. Renčo M, Sasanelli N, Papajová I and Maistrello L, Nematicidal effect of chestnut tannin solutions on the potato cyst nematode *Globodera rostochiensis* (Woll.) Barhens. *Helminthologia* **49**: 108-114 (2012).

7. Riga E, Topp E, Potter J, Welacky T, Anderson T and Tenuta A, The impact of plant residues on the soybean cyst nematode, *Heterodera glycines*. *Can J Plant Pathol* **23**: 168-173 (2001).

8. Charlson D and Tylka G, *Heterodera glycines* cyst components and surface disinfestants affect *H. glycines* hatching. *J Nematol* **35**: 458-464 (2003).

9. Tanino K, Takahashi M, Tomata Y, Tokura H, Uehara T, Narabu T, et al., Total synthesis of solanoeclepin A. *Nat Chem* **3**: 484 (2011).

10. Jones P, Byrne J and Devine K, *In vitro* studies on the relative availability and mobility in soil of natural hatching factors for the potato cyst nematodes, *Globodera rostochiensis* and *G. pallida*. *J Nematol* **3**: 75-83 (2001).

11. Ochola JA, Cortada L, Ng’ang’a M, Hassanali A, Coyne D and Torto B, Mediation of potato-potato cyst nematode, *G. rostochiensis* interaction by specific root exudate compounds. *Front Plant Sci.* DOI: 10.3389/fpls.2020.00649 **(**2020)

12. Pridannikov MV, Petelina GG, Palchuk MV, Masler EP and Dzhavakhiya VG, Influence of components of *Globodera rostochiensis* cysts on the *in vitro* hatch of second-stage juveniles. *J Nematol* **9**: 837-844 (2007).

13. Nour SM, Lawrence JR, Zhu H, Swerhone GD, Welsh M, Welacky TW, et al., Bacteria associated with cysts of the soybean cyst nematode (*Heterodera glycines*). *App Environ Microbiol* 69: 607-615 (2003).

14. Torto B, Cortada L, Murungi LK, Haukeland S and Coyne DL, Management of cyst and root knot nematodes: A chemical ecology perspective. *J. Agric. Food Chem* **66**: 8672-8678 (2018).

15. Scholte K, Growth and development of plants with potential for use as trap crops for potato cyst nematodes and their effects on the numbers of juveniles in cysts. *Ann Appl Biol* **137**: 31-42 (2000)

16. Timmermans B, Vos J, Van Nieuwburg J, Stomph T, Van der Putten P and Molendijk P, Field performance of *Solanum sisymbriifolium*, a trap crop for potato cyst nematodes. I. dry matter accumulation in relation to sowing time, location, season and plant density. *Ann Appl Biol* **150**: 89-97 (2007).

17. Sasaki-Crawley A, Signalling and behaviour of *Globodera pallida* in the rhizosphere of the trap crop *Solanum sisymbriifolium*. *Asp. Appl. Biol.* **103**: 45-51 (2013).

18. Hoysted GA, Bell CA, Lilley CJ and Urwin PE, Aphid colonization affects potato root exudate composition and the hatching of a soil borne pathogen. *Front Plant Sci* **9**: 1278 (2018).

19. Zhou L, Wang J, Wang K, Xu J, Zhao J, Shan T, et al. Secondary metabolites with antinematodal activity from higher plants, in Studies in Natural Products Chemistry, ed. by Atta-ur-Rahman, Elsevier, pp. 67-114 (2012).

20. Wang YS, A ; Zhao, YS ; Zhao, J ; Liu, D ; Zhu, XF ; Liu, XY ; Fan, HY ; Chen, LJ ; Duan, YX Effect of culture filtrate of *Sinorhizobium fredii* sneb183 on the activity and behavior of soybean cyst nematode (*Heterodera glycines* ichinohe, 1952). *Appl Ecol Env Res* **18**: 1129-1140 (2020).

21. Yu Q, Tsao R, Chiba M and Potter J, Selective nematicidal activity of allyl isothiocyanate. *J Food Agric Environ* **3**: 218-221 (2005).

22. Ghisalberti EL. Secondary metabolites with antinematodal activity, in Studies in Natural Products Chemistry, ed. by Atta-ur-Rahman Elsevier, pp. 425-506 (2002).

23. Díaz-Tielas C, Graña E, Reigosa M and Sánchez-Moreiras A, Biological activities and novel applications of chalcones*. Planta Daninha* **34**: 607-616 (2016).

24. Silva FJ, Campos VP, Oliveira DF, Gomes VA, Barros AF, Din ZU, et al., Chalcone analogues: Synthesis, activity against *Meloidogyne incognita*, and in silico interaction with cytochrome P450. *J Phytopathol* **167**: 197-208 (2019).

25. Tian H, Riggs RD and Crippen DL, Control of soybean cyst nematode by chitinolytic bacteria with chitin substrate*. J Nematol* **32**: 370 (2000).

26. Perry R, Warrior P, Kerry B and Twomey U, Effects of the biological nematicide, DiTera, on hatching of *Globodera rostochiensis* and *G. pallida*. *J* *Nematol* **2**: 355-362 (2000).

27. Cox D, Dyer S, Weir R, Cheseto X, Sturrock M, Coyne D, et al., ABC transporters alter plant-microbe-parasite interactions in the rhizosphere. *Sci Rep*: 19899 (2019).

28. Claire W, David MK and Julia MC, Allyl isothiocyanate shows promise as a naturally produced suppressant of the potato cyst nematode, *Globodera pallida*, in biofumigation systems. *J Nematol* **19**: 389-402 (2017).

29. Perry R. Hatching, in The Biology of Nematodes, ed. by Lee DL, CRC Press, London and New York, pp 294-337 (2002).

30. Zhang J, Li Y, Yuan H, Sun B and Li H, Biological control of the cereal cyst nematode (*Heterodera filipjevi*) by *Achromobacter xylosoxidans* isolate 09X01 and *Bacillus cereus* isolate 09B18. *Biol Control* **92**: 1-6 (2016).

31. Wang C, Masler EP and Rogers ST, Responses of *Heterodera glycines* and *Meloidogyne incognita* infective juveniles to root tissues, root exudates, and root extracts from three plant species. *Plant Dis*  **102**: 1733-1740 (2018).

32. Kammerhofer N, Radakovic Z, Regis JMA, Dobrev P, Vankova R, Grundler FMW, et al., Role of stress-related hormones in plant defence during early infection of the cyst nematode *Heterodera schachtii* in *Arabidopsis*. *New Phytol* **207**: 778-789 (2015).

33. Būda V and Čepulytė-Rakauskienė R, The effects of α-solanine and zinc sulphate on the behaviour of potato cyst nematodes *Globodera rostochiensis* and *G. pallida*. *J Nematol* **17**: 1105-1111 (2015).

34. Devine KJ and Jones PW, Investigations into the chemoattraction of the potato cyst nematodes *Globodera rostochiensis* and *G. pallida* towards fractionated potato root leachate. *J Nematol* **5**: 65-75 (2003).

35. Būda V and ČepulytĖ-RakauskienĖ R, The effect of linalool on second-stage juveniles of the potato cyst nematodes *Globodera rostochiensis* and *Globodera pallida*. *J Nematol*  **43**: 149 (2011).

36. Fleming TR, Maule AG and Fleming CC, Chemosensory responses of plant parasitic nematodes to selected phytochemicals reveal long-term habituation traits. *J Nematol*  **49**: 462-471 (2017).

37. Farnier K, Bengtsson M, Becher PG, Witzell J, Witzgall P and Manduríc S, Novel bioassay demonstrates attraction of the white potato cyst nematode *Globodera pallida* (Stone) to non-volatile and volatile host plant cues. *J Chem Ecol* **38**: 795-801 (2012).

38. Hu Y, You J, Li C, Pan F and Wang C, Assessing the effects of water extract of *Narcissus tazetta* bulb on hatching, mortality, chemotaxis and reproduction of the soybean cyst nematode, *Heterodera glycines*. *J Nematol* **22**: 53-62 (2019).

39. Hu Y, You J, Li C, Williamson VM and Wang C, Ethylene response pathway modulates attractiveness of plant roots to soybean cyst nematode *Heterodera glycines*. *Sci Rep* **7**: 41282 (2017).

40. Escudero Martinez CM, Guarneri N, Overmars H, van Schaik C, Bouwmeester H, Ruyter-Spira C, et al., Distinct roles for strigolactones in cyst nematode parasitism of Arabidopsis roots. *Eur J Plant Pathol* **154**: 129-140 (2019).
